# Signs, meanings and practices of people living with human t-cell lymphotropic virus type 1 or tropical spastic myelopathy

**DOI:** 10.1186/s41687-020-00198-6

**Published:** 2020-05-04

**Authors:** Genildes Oliveira Santana, Ana Mary Libório, Ana Verena Galvão, Milena Pereira Pondé, Katia Nunes Sá

**Affiliations:** 1 Department of Post Graduate, Bahiana School of Medicine and Public Health, Salvador, Bahia Brazil; 2 Multidisciplinary Reference Center for Assistance and Research for Family and Patients with HTLV the Assisting Teaching the Bahia School of Medicine and Public Health, Salvador, Bahia Brazil

**Keywords:** Tropical spastic paraparesis, Human T-lymphotropic virus 1, Qualitative research, Psychosocial approach

## Abstract

**Background:**

Human T-cell lymphotropic virus type 1 (HTLV-1) spreads silently in the world’s population and causes several syndromes. Among these, HTLV-1 associated myelopathy, also called tropical spastic paraparesis (HAM/TSP), affects the nervous system. It causes sensorimotor losses, spasticity, muscle weakness, voiding and sexual dysfunction, pain, and balance disorders. There is limited knowledge of the feelings, experiences, and coping mechanisms associated with this neglected disease. The objective of the present qualitative study was to investigate the signs, meanings, and practices of people with HAM/TSP, through narratives obtained from focus groups and individual semi-structured face-to-face interviews.

**Results:**

Thirty-eight individuals diagnosed with HAM/TSP participated in the study. The following categories and subcategories emerged from the participants: Signs—physical signs, symptoms, and discovery of the disease; Meanings—reaction to diagnosis and knowledge of disease, fears, and expectations; Practices—daily life, leisure, religious, and treatment activities.

**Conclusions:**

People with HAM/TSP suffer from symptoms that limit their social participation, and they are affected by complex and multidimensional feelings. This awareness can contribute to the implementation of public policies—focused on the real perspective of these patients—that provide more directed, empathic, and harmonious care for these individuals.

## Introduction

Human T-cell lymphotropic virus type 1 (HTLV-1) was the first retrovirus identified in humans [1]. Its infection occurs either vertically (breastfeeding) or horizontally (through sexual intercourse, blood transfusion, or contamination with piercing objects) [2]. It is estimated that there are 5 to 10 million infected people worldwide [3], of whom more than 2 million are in Brazil [4]. Salvador is the city with the highest seroprevalence in the country, with 1.76% of the population affected. The majority of infected people are female with black skin color and low socioeconomic status [5].

Approximately 3–5% of HTLV-1-infected people develop HTLV-1 associated myelopathy or tropical spastic paraparesis (HAM/TSP). It is a progressive neurodegenerative condition that predominantly affects the spinal cord and causes chronic pain, postural imbalance, sphincter and sexual disorders, and gait and balance alterations, all of which negatively impact quality of life [6, 7]. Five to ten years after HAM/TSP diagnosis, people need some walking aid [6].

The approach to this health condition has been elaborated based on anatomopathological, clinical, and functional quantitative markers [8]. Similarly, the efficacy of pharmacological and physiotherapeutic interventions is tested by evaluating quantifiable outcomes [9–12]. Listening to the “voices” of people who live with HAM/TSP, with regards to the symptoms, perceptions, feelings, perspectives, and ways of coping, can help to uncover questions that quantitative research does not achieve. Indeed, although much is known about the biomedical aspects of HAM/TSP, little is known about the effect of the disease and therapeutic protocols on psychosocial parameters [13]. Assessing the perception of affected people can reveal subjective aspects that affect the results of therapeutic interventions and clinical evolution. It can also help establish grounds for health actions recommended by the World Health Organization (WHO) for neglected diseases [14].

Qualitative research aims to gather systematic knowledge on the ways of thinking and acting for specific populations. The analysis of signal systems, meanings and practices is grounded in the study of patients’ narratives about their experience with a disease. Based on interpretative anthropology, this analytical model investigates the subjective experience of illness and treatment in different sociocultural contexts [15]. Thus, the main objective of the present study was to investigate the signs of the disease process, meanings attributed to the experience and treatments of the disease, and reactive and self-care practices, expressed in the cultural networks of people living with HAM/TSP.

## Methods

### Study design and participants

This study was a descriptive and exploratory examination with a qualitative approach. The study sought to systematize narratives from participants via focus groups (FGs) and face-to-face semi-structured interviews. Information analysis was based on the system of culturally constructed signs, meanings, and practices. It was conducted at the Advanced Physiotherapy Clinics (CAFIS) of the Teaching Assistance Ambulatory, Bahiana School of Medicine and Public Health, located in Salvador, Bahia, Brazil. The population was comprised of patients diagnosed with HAM/TSP who were enrolled in the Integrative and Multidisciplinary Centre for Assistance and Research to Family and HTLV Carriers (CHTLV) of the Bahiana School of Medicine and Public Health in 2014.

### Recruitment and sampling

Enrollment in the study was intentional and voluntary; it occurred via an invitation that was presented to CHTLV participants who were participating in another research project [16]. Fifty-six people who were part of the clinical trial were invited to participate in the present study. Thus, 38 individuals were included in the final sample. They were distributed in the FGs, represented by the code G, or the interviews, represented by the code E. The groups were still coded by the participants with the letter P and numbered from 1 to 38 (which represented each participant).

Participants were included if they had a confirmed diagnosis of HTLV-1 infection by enzyme-linked immunosorbent assay (ELISA) and western blot exam, clinical diagnosis for HAM/TSP according to Castro-Costa et al. criteria [17], were enrolled in CHTLV, and were above 18 years of age. Patients were excluded if they had difficulties understanding questions or communicating.

### Procedures

Data collection was performed by a researcher with experience in the FG and semi-structured interview techniques. The main researcher (the facilitator) is a physiotherapist, a master in Human Development and Social Responsibility for 25 years with 32 years of clinical practice. The facilitator was accompanied by two undergraduate students from the Physical Therapy course at the Bahiana School of Medicine and Public Health. The students were trained by the researcher. One served as an observer, responsible for making notes of aspects observed in the dynamics of the FG, and the other undergraduate student was responsible for audio recordings. The researcher spoke in person to the participants to invite them to a FG. On the eve of each FG, the researcher confirmed the time and place of the meeting via telephone.

A total of 11 FGs were created, out of which 6 were categorized as pre-training, meaning they took place after the first evaluation for the RTC. These 6 FGs were constituted as follows: FG1 (1 man and 4 women); FG2 (2 men and 3 woman); FG3 (2 men and 1 woman); FG4 (4 men and 4 women); FG5 (2 men and 4 women); FG6 (2 men and 3 women). Some participants reports that they had no time, transportation, company, or financial availability or they had other commitments to medical consultations and/or treatments at the same time as the survey. After training and the last evaluation at the ECR, 13 participants of the pre-training group attended the 5 post-training FG in addition to 3 new participants. These groups were formed as follows: FG7 (1 man and 3 women); FG8 (3 women); FG9 (2 men and 1 woman); FG10 (2 men and 1 woman) and FG 11 (3 women). FGs were performed for approximately 1.5 h.

FGs are group discussions, organized to explore a specific set of issues, where participants can interact, explore each other’s arguments, and express topics they consider important [18].. It also provides the interpretation of beliefs, values, concepts, conflicts, confrontations, and points of view [19]. These features are why it was one of the techniques selected for this research.

The FG environment should be pleasant, comfortable, and warm. Thus, we opted for the use of incense, which lightly perfumed the room, relaxing music with sounds of water and nature, and snacks throughout the meeting [20]. Each FG was conducted in a private setting in the physiotherapy clinic (CAFIS). The location of the room allowed the meetings to proceed without external interference. This design facilitated the debate and ensured privacy, comfort, easy access, and a neutral environment [21]. The availability of the chairs in a circle allowed face-to-face interaction, good eye contact, and the same field of view for everyone [21]. After clarifying the objectives of the study and providing written informed consent, the participants were invited to relax and the procedures recommended by Iervolino and Pelicione [22] were adopted. Relaxation and integration techniques were used prior to the application of the method. Semi-structured interviews were conducted in a private, air-conditioned room at the CAFIS. The guide for the questions used in the FG and interviews are described below.

### Guiding questions of the pre-treatment FG

How did you discover the disease?

How did you get the diagnosis?

What were the symptoms?

What is the relationship of exercises to your health problem?

What do you expect from participation in this project?

What reasons could prevent your participation in this project?

### Guiding questions for post-treatment FG

Were your expectations reached?

How was your participation?

Did you comply with the protocol?

Did you notice changes in your condition?

Semi-structured interviews were also performed with 12 participants, out of which 8 were FG participants and 4 new members that participated in the RCT control group. 7 men and 5 women were interviewed. Twelve interviews were sufficient to reach data saturation because the information generated in the last interviews was repeated and no new code emerged. The average duration of the interviews was approximately 1 h. Semi-structured interviews were conducted in a private, air-conditioned room at the CAFIS. Semi-structured interview questions: How is your day-to-day activity? How do you do your exercise as part of your day-to-day routine?

### Analytical approach

The analysis of the systems of signs, meanings and practices, investigates experiences of the illness and healing process in different socio-cultural contexts [15]. After the focus group transcripts, interviews, and field notes were carried out by the main researcher, the transcribed information was read and reread by 2 researchers separately, thus increasing reliability. Content analysis techniques were performed manually to detect units of meaning and nexus categories. After identifying the codes, the authors met to discuss the findings of the themes related to the signs (category refers to symptoms that indicate the disease or suffering described in the patients’ narratives), meanings (category refers to how the person perceives the problem and how s/he constructs, in their imagination, the received diagnosis), and practices (category refers to the creation of strategies to solve a specific problem of myelopathy). After the data was revised multiple times and a consensus was reached, the findings were compiled, and a matrix describing the most representative themes was created in the results section.

## Results

The sample consisted of 38 people with HAM/TSP; the mean age was 54.2 ± 10.28 years. The majority of participants were women (57.89%), of African descent (47.37%), married (42.10%), had completed primary schooling (36.84%), and of the average socioeconomic class (55. 26%). The mean disease duration was 11.45 ± 8.31 years and mean body mass index (BMI) was 24.67 ± 3.21 kg/m2. Twenty (52.64%) participants did not practice physiotherapy regularly and 23 (60.53%) used a walking or gait devices (Table 1).

**Table 1:** Sociodemographic and clinical characteristics of individuals with HAM/TSP of the CTHTLV of the Bahiana School of Medicine and Public Health, Salvador, Bahia, Brazil

Variables	*N* = 38	N (%)
Age, years (M ± SD)		54.20 ± 10.28
Sex	Female	22 (57.89)
	Male	16 (42.11)
Skin color	Black	18 (47.37)
	Mixed race	17 (44.73)
	White	3 (7.9)
	Yellow	0 (0)
Socioeconomic class	A	1 (2.63)
	B	3 (7.89)
	C	21 (55.26)
	D	12 (31.59)
	E	1 (2.63)
Marital status	Single	10 (26.32)
	Married	16 (42.10)
	Separated	3 (7.89)
	Widowed	9 (23.68)
Education Level	Illiterate	1 (2.63)
	Elementary School Incomplete	10 (26.32)
	Elementary School complete	14 (36.84)
	Secondary Education	7 (18.42)
	Higher Education	6 (15.79)
Walking or gait devices	Does not use	15 (39.47)
	Uses	23 (60.53)
Physiotherapy	Yes	18 (47.36)
	No	20 (52.64)
Disease duration, years (M ± SD)		11.45 ± 8.31
BMI, kg/m^2^ (M ± SD)		24.67 ± 3.21

*M* Mean, *SD* Standard deviation; Social Class A and B = high, C = average; D and E = low (parameters IBGE, Brazil); *BMI* Body mass index

**Fig. 1:**
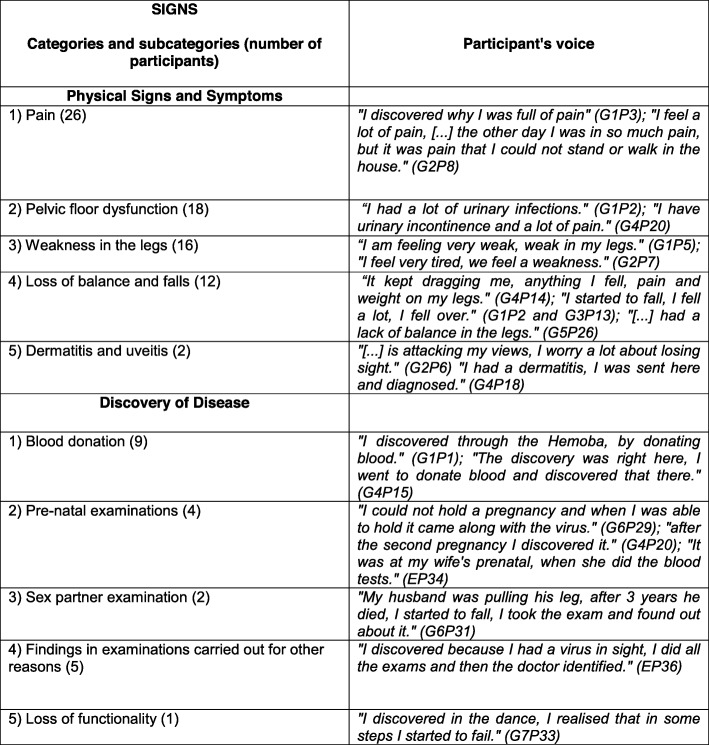
Signs—Category and subcategories

**Fig. 2:**
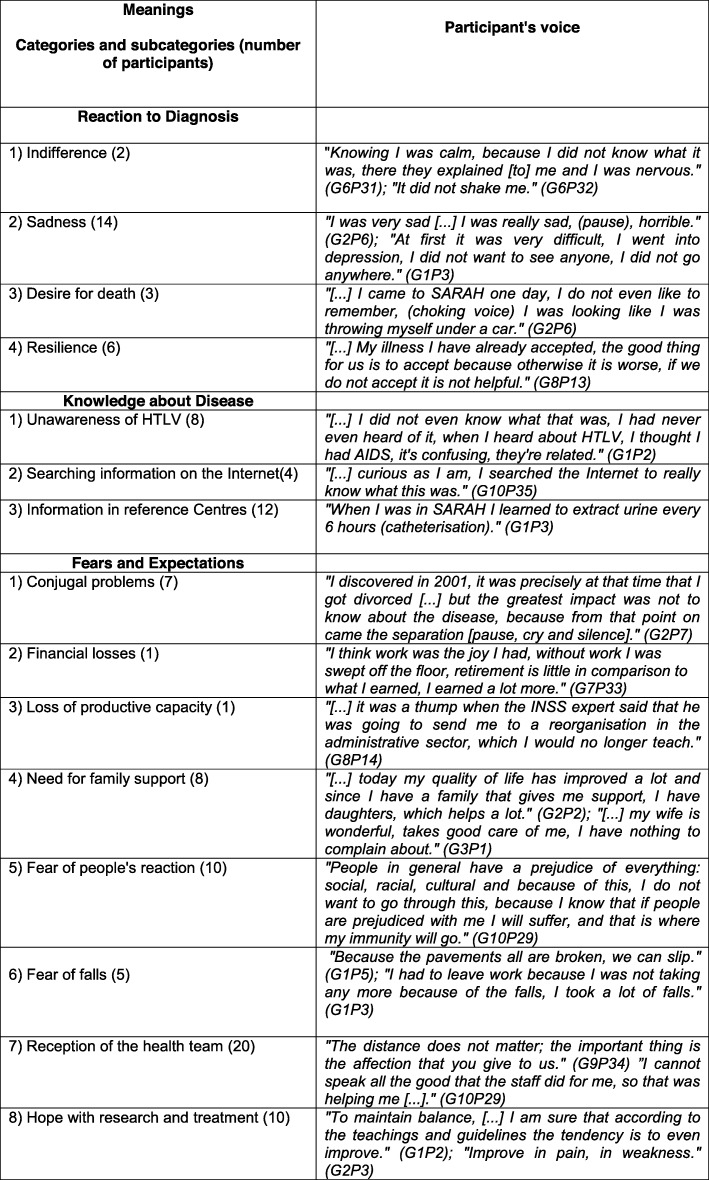
Meaning—Category and subcategories

**Fig. 3:**
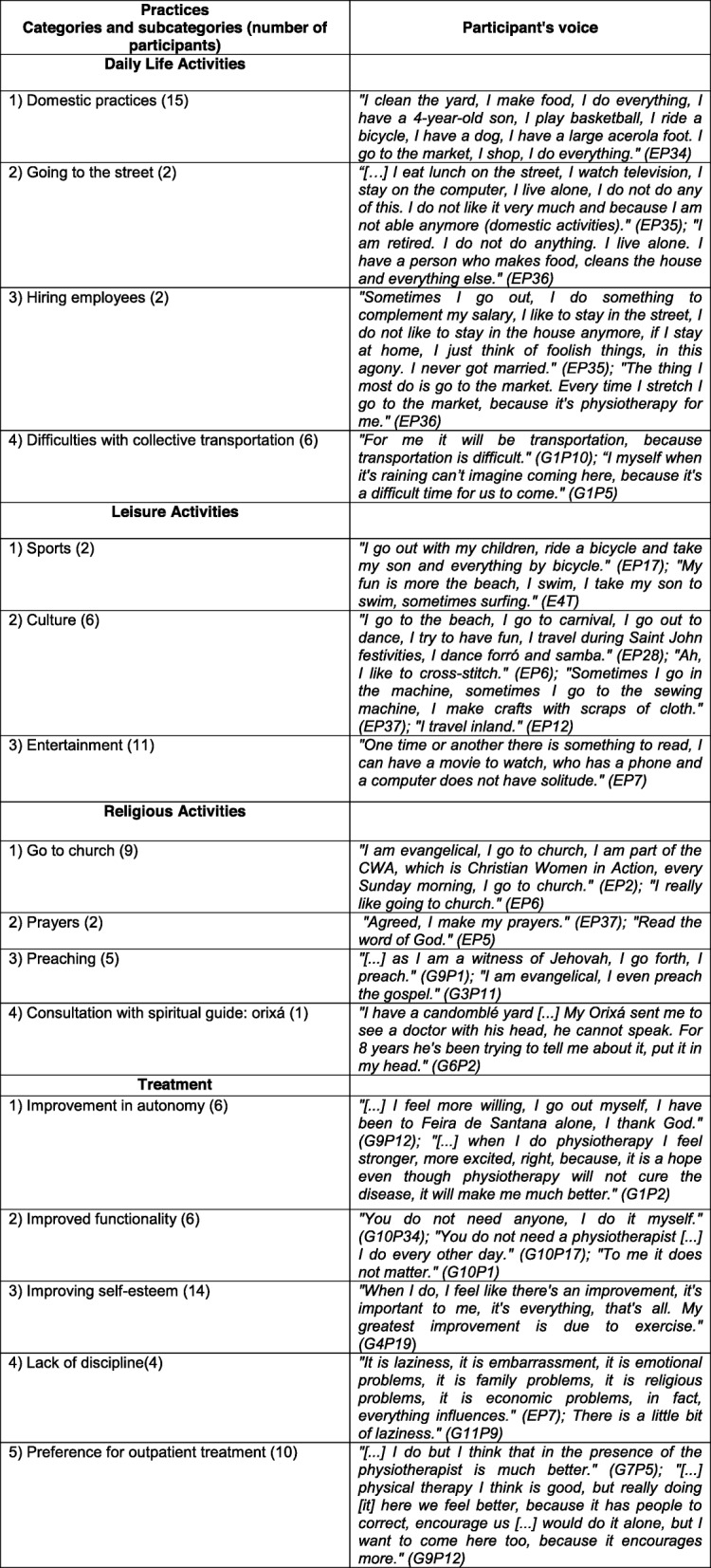
Practices—Category and subcategories

From the analyses of the information collected in the FGs and interviews, the following categories emerged: Signs—physical signs, symptoms, and discovery of the disease (Fig. 1); Meanings—reaction to the diagnosis, knowledge about the disease, fears and expectations (Fig. 2); Practices—activities of daily living, leisure activities, religious activities, and treatment (Fig. 3).

### Signs

The Signs category refers to symptoms that indicate the disease or suffering described in the patients’ narratives (Fig. 1).

Most participants shared the same symptoms of HAM/TSP, especially pain, pelvic floor dysfunction, weakness in the legs that led to loss of balance and falls. In the present study, dermatitis and uveitis were mentioned by only 1 participant each. Regarding discovery of the disease, most participants discovered the infection through blood donation, followed by prenatal examination and sexual partner examination. Loss of functionality also led patients to seek medical services.

### Meanings

The category Meanings refers to how the person perceives the problem and how s/he constructs, in their imagination, the received diagnosis.

Fourteen respondents reported feelings of sadness and depression upon receiving the HAM/TSP diagnosis. Three patients felt the desire to die, feelings that demonstrate suffering and anguish permeate the imagination of these people. In contrast, 6 participants adopted a resilient stance as a way to adapt to change and overcome adversity.

Among the fears and expectations, most respondents cited marital problems, financial loss, loss of productive capacity, need for family support, fear of people’s reaction, and fear of falling. They also welcomed the potential benefits of health care and expressed hope about research and treatment. We observed the presence of family support, mainly from mothers and wives, in most of the narratives. However, in some cases the support came from strangers.

### Practices

The Practices category refers to the creation of strategies to solve a specific problem of myelopathy.

Regarding the practices of the exercise programme, all respondents reported greater autonomy and balance. Obstacles such as lack of self-discipline, financial problems, low levels of education, walking difficulties, and cultural factors make it tough to adhere to exercise protocols. Ten participants indicated a preference for performing exercises in the clinic, even though they knew how to do the exercises alone. According to them, in addition to socialization, working in the clinic increases their confidence in safely, correctly executing the exercises. There is a need for multidisciplinary rehabilitation centers for outpatient treatment of individuals with HAM/TSP that are comprised of professionals prepared to promote care for this population.

Nine interviewees indicated that going to church, regardless of religion, is an act of exercise of faith and socialization. Preaching was also cited by 5 patients. Praying was another way of contacting the Divine. Only 1 interviewee, of the African religion Candonblé, reported talking to his orixa as a way of exercising faith and asking for problem-solving advice.

## Discussion

### General aspects of the sample

The sociodemographic characteristics of the participants are similar to prior reports, in which most of the samples were composed of women [2, 3]. Our data further confirmed that the population most affected by HAM/TSP has lower education and socioeconomic levels, is of African descent, and is married [4]. Most of the participants were middle-aged adults, which suggests a period of latency of the virus for several decades [6].

The median time of clinical manifestations varied from months to decades, which is a finding consistent with previous studies [4]. The use a walking or gait devices was present in more than half of the participants. This data highlights the pronounced functional disability, as reported in previous studies [12, 23]. Despite this incapacity, the majority of the interviewees did not perform physiotherapy, a finding similar to a previous study [10]. The benefits of rehabilitation programs for this population are evident [9–11]. In the arsenal of techniques employed by physiotherapists, exercises are the main modality used to improve functional capacity and quality of life. The positive effects of home exercise programs underscore the prospect of assisting this population in today’s contexts [24].

### Signs

The highest reported symptom was daily pain, especially in the lower back and lower limbs [7, 8], which is data consistent with previous studies [24–26]. Pain is the principal complaint of those affected by the HAM/TSP and causes significant loss of quality of life [27]. Pelvic floor dysfunction was also reported by participants, especially women [28, 29]. This situation interferes with social life because loss of urine, unpleasant odor, and need for absorbents indicates the fragility of these people. Additionally, the urgent need to urinate several times at night interferes with sleep quality and impairs sexual performance, self-care, and the willingness to perform various activities, all of which reduce self-esteem and self-confidence [30].

Weakness in the legs, loss of balance, and falls were also cited as frequent symptoms according to related literature [7]. The risk of falling is a public health problem because the consequences bring great suffering and increased expenses resulting from surgeries and hospitalization [31, 32]. These issues cause more withdrawal social behavior and decrease the ability to perform day-to-day work and activities that were previously executed. Furthermore, dermatosis has been documented with a high frequency in symptomatic [33] and asymptomatic individuals [8, 34]. Genital candidiasis, associated in most cases with urinary incontinence [35], was also reported. The association between ocular manifestations and people infected with HTLV-1 has also been reported [36]. Although the literature evidences the presence of dermatosis and eye problems in patients with HAM/TSP, these symptoms were rarely reported in the present study.

The discovery of the disease was casual in general. These findings indicate the need to include ELISAs in routine basic care for the early detection of HTLV-1 in endemic areas, such as Salvador [5]. Blood banks have been the main detection site, thanks to Ordinance No. 1376 of the Ministry of Health [37]. The prohibitive cost of testing is a limiting factor for screening in economically disadvantaged countries [38]. A study conducted in Japan showed that through public policy actions, with the insertion of serological testing in pregnant women and interruption of breastfeeding of seropositive women, vertical transmission was reduced from 20 to 3% [39].

### Meaning

Most respondents shared the presence of mental disorders, in the face of disease progression, as well as loss of the ability to work and enjoy leisure time. The reaction to the diagnosis corresponds to the stages of mourning described by Elizabeth Kubler Ross [40]. Acceptance of the disease does not occur immediately after diagnosis. Untroubled acceptance will usually only occur after decades, when the patients become more adherent to the therapeutic programs [41].

Health professionals and society in general have a profound lack of knowledge about HTLV infection, and this knowledge gap has implications for both diagnosis and care practice. The fact that only 1–5% of the infected develop symptoms makes it difficult to implement public policies aimed at this population and perpetuates the spread of the virus [8]. Indeed, HTLV is an “invisible” virus; its spread generally occurs silently in the population [41]. Coping with the disease should be approached in the biopsychospiritual model recommended by the WHO [42], namely by a team aware of the psychological stages expected after the diagnosis and who can guide the minimization of adverse reactions. The rise of the Internet has favored health literacy [43]. However, the number of websites that do not guarantee quality information is still significant. Only limited content is available on the HTLV-1.

Health centers are sought in the hope of finding other more effective treatments with the hope that one day a cure will be discovered [44]. HTLV-1 is a sexually transmitted disease, thus often affecting marital relationships. Suffering often stems from a sense of betrayal due to a partner’s extramarital affairs and/or a lack of encouragement in seeking new partners due to fears of infecting others. Issues include loss of libido, perineal hypersensitivity, vaginismus, pelvic floor hypertonia caused by neuromotor changes, and impaired sexual activities and pleasure [30]. In addition, premature menopause is common in infected women [28]. This complex scenario reduces the quality of life. Socio-educational actions are fundamental for the practice of protected sex and reduction of the number of sexual partners that increase the risks of contamination.

Another factor involved in chronic diseases is the commitment of financial resources of patients and their families. The transition from productive worker to retired due to disability markedly reduces wages and consequently lowers the socioeconomic status [45]. The most affected individuals are frequently the family providers; changes in social roles become necessary. In addition to a reduction in the ability to provide for her/his family, symptomatic people often become dependent and vulnerable [12]. Spending on diapers, transportation, and exams increases. These purchases, coupled with reduced income, require substantial family rearrangements and decrease the purchasing power of all involved.

Loss of days at work, medical leave, and early retirement generate high costs for the entire society [45]. The subjects often feel unable to contribute socially and feel shame and fear of bothering others, which generates great suffering and social exclusion. The families undergo major changes in their composition [46]. They are the primary source of support for HTLV-1-seropositive people. The family should seek support, security, affection, and respect, all of which are fundamental elements for better coping.

### Practices

Individual or group exercise programs, at the clinic or at home, are recognized as useful for improving function in this population [9–11]. However, difficulties with self-discipline limit the implementation of home programs in Brazil [10**].**

The chronic degenerative evolution of HAM/TSP leads to the need for the use of walking aids, such as canes, walkers, and wheelchairs and total dependence on care from others in the final phase of the disease [23, 27]. This condition reduces recreational and sports activities, which lead to physical inactivity and increase the risk of comorbidities [31]. Cell phones and computers were reported as important forms of leisure.

It is also clear that spiritual practice affects the health of people with HTLV. Although religious diversity in Brazil demonstrates that the country has become secular, the search for a religious meaning for practical experience is fundamental to addressing health problems. In the present study, most women sought to exercise their faith in various ways while men were in the minority. Instead, they chose to preach. Spirituality is part of the constitution of all men, regardless of any spiritual experience [47].

### Implications for clinical practice and future research

This study is the first qualitative research that involves people with HAM/TSP, assisted at CTHTLV. The knowledge gained from this study by authorities and health professionals may promote greater understanding of the needs that permeate this population. This awareness can contribute to the implementation of public policies, focused on the real perspective of these patients, through more targeted care that is empathic and harmonious for these individuals.

Future research should aim to solve the problems presented in the current study, including accessibility, public policies, knowledge about the virus, physiotherapy, and leisure, among others.

## Conclusions

It can be concluded that people with HAM/TSP suffer from symptoms that are limiting their social participation. They are affected by complex and multidimensional feelings. Activities of daily living, including leisure, spiritual, and therapeutic assistance, help in coping with this neglected health condition. Public policies should be implemented to allow an integrated multidisciplinary team to minimize the effects of this infection on the quality of life of these people.

## Data Availability

http://www7.bahiana.edu.br//jspui/handle/bahiana/2919

## Abbreviations

ADAB: Assisting teaching ambulatory
CTHTLV: Integrative and Multidisciplinary Center for Assistance and Research to Family and HTLV Carriers of the Bahiana School, Salvador
CAFIS: Advanced physiotherapy clinic
EBMSP: Bahia School of Medicine and Public Health
ELISA: Enzyme-linked immunosorbent assay
HAM/TSP: Human T-cell lymphotropic virus type 1 associated myelopathy/tropical spastic paraparesis
HEMOBA: Bahia State Hematology and Hemotherapy Foundation
HTLV-1: Human T-cell lymphotropic virus type 1
RCT: Randomized clinical trial
SARAH: Network of rehabilitation hospitals
WHO: World Health Organization
